# Abnormal brain functional networks in end‐stage renal disease patients with cognitive impairment

**DOI:** 10.1002/brb3.2076

**Published:** 2021-02-19

**Authors:** Zheng Yue, Pengming Wang, Xuekun Li, Jipeng Ren, Baolin Wu

**Affiliations:** ^1^ Department of Magnetic Resonance The First Affiliated Hospital of Xinxiang Medical University Weihui China; ^2^ Department of Radiology The First Affiliated Hospital of Xinxiang Medical University Weihui China; ^3^ Department of Radiology Huaxi MR Research Center (HMRRC) Functional and Molecular Imaging Key Laboratory of Sichuan Province West China Hospital of Sichuan University Chengdu China

**Keywords:** brain functional networks, cognitive impairment, end‐stage renal disease, functional magnetic resonance imaging, graph theoretical analysis, resting state

## Abstract

**Introduction:**

Cognitive impairment (CI) is common in patients with end‐stage renal disease (ESRD). Neuroimaging studies have demonstrated structural and functional brain alterations underlying CI in patients with ESRD. However, the patterns of change in whole‐brain functional networks in ESRD patients with CI remain poorly understood.

**Methods:**

We enrolled 66 patients with ESRD (36 patients with CI and 30 patients without CI) and 48 healthy control subjects (HCs). We calculated the topological properties using a graph theoretical analysis. An analysis of covariance (ANCOVA) was used to compare network metrics among the three groups. Moreover, we analyzed the relationships between altered network measures and clinical variables in ESRD patients with CI.

**Results:**

Compared with HCs, both patient groups showed lower local efficiency and small‐worldness. ESRD patients had decreased nodal centralities in the default mode regions and right amygdala. Comparison of the two patient groups showed significantly decreased global (small‐worldness) and nodal (nodal centralities in the default mode regions) properties in the CI group. Altered nodal centralities in the bilateral medial part of the superior frontal gyrus, left posterior cingulate gyrus, and right precuneus were associated with cognitive performance in the CI group.

**Conclusion:**

Disrupted brain functional networks were demonstrated in patients with ESRD, which were more severe in those with CI. Moreover, impaired nodal centralities in the default mode regions might underlie CI in patients with ESRD.

## Graphical abstract

The present study aimed to investigate the patterns of change in brain functional networks in end‐stage renal disease (ESRD) patients with cognitive impairment (CI). Disrupted brain functional networks were identified in patients with ESRD, which were more severe in those with CI. Altered nodal centralities in the default mode regions might be the neural basis of CI in ESRD patients.

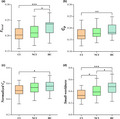

## 1 INTRODUCTION

Cognitive impairment (CI) is common in patients with end‐stage renal disease (ESRD), especially in those receiving hemodialysis (HD). Approximately 30% to 60% of ESRD patients who are undergoing HD exhibit CI (Bugnicourt et al., 2013), including the deficits in executive function, memory, attention, and motor performance (Jung et al., 2013; Kalirao et al., 2011; Pereira et al., 2005). At present, the pathophysiological mechanisms of CI in patients with ESRD remain unclear. Therefore, it is urgent to explore potential imaging biomarkers for a better understanding of the pathophysiological mechanisms underlying CI in patients with ESRD, which is particularly important for the early diagnosis, treatment, and intervention.

Neuroimaging techniques have provided valuable tools to explore potential imaging biomarkers for ESRD‐related neurological complications. For example, previous studies with voxel‐based morphometry, surface‐based morphometry, and diffusion tensor imaging (DTI) revealed gray matter volume deficits (Qiu et al., 2014; L. J. Zhang et al., 2013), decreased cortical thickness (Dong et al., 2018), and reduced white matter integrity (Drew et al., 2017; R. Zhang et al., 2015) in patients with ESRD. In addition, cerebral metabolic and functional abnormalities were demonstrated in ESRD patients who underwent HD using different analyses, including arterial spin labeling (Jiang et al., 2016), magnetic resonance spectroscopy (C. Y. Zhang et al., 2017), and single‐photon emission tomography (Polinder‐Bos et al., 2020).

In recent years, studies using resting‐state functional magnetic resonance imaging (Rs‐fMRI) have attempted to detect the brain functional alterations underlying neuropsychological impairments in patients with ESRD (Luo et al., 2016; Ni et al., 2014). Prior Rs‐fMRI studies have revealed that ESRD patients exhibited abnormal intrinsic brain activity and disrupted intra‐ and interregional functional connectivity, especially in brain regions associated with the default mode network (DMN) (Liang et al., 2013; Luo et al., 2016; Ni et al., 2014; Zheng et al., 2014). However, whole‐brain functional network alterations underlying cognitive declines in patients with ESRD have not been fully understood.

Graph theoretical analysis models the human brain as a complex network represented graphically by a collection of nodes and edges, which can be defined as anatomical elements (e.g., brain regions) and relationships between these regions (e.g., connectivity), respectively (J. Wang et al., 2010). This method provides a useful tool to identify alterations in the topological organization of brain networks in various clinical populations. Recent studies have demonstrated altered structural and functional brain networks in patients with ESRD (Chou et al., 2019; Jin et al., 2020; Park et al., 2020). However, authors of these studies did not focus on characterizing the topological organization of whole‐brain functional networks in ESRD patients with CI. To gain our knowledge of the pathophysiological mechanisms underlying CI in ESRD patients, we aimed to conduct a prospective study to investigate the patterns of change in the topological organization of brain functional networks in ESRD patients with CI.

In our study, we hypothesized that (a) small‐world properties should be demonstrated in ESRD patients with and without CI and healthy controls (HCs); (b) both ESRD patients with and without CI should demonstrate impaired whole‐brain functional networks in global and local levels; (c) ESRD patients with CI, relative to those without CI, should demonstrate a more severe disruption of whole‐brain functional networks; and (d) some altered network measures may correlate with cognitive function of patients with ESRD. To test our hypotheses, we included 36 ESRD patients with CI and 30 ESRD patients without CI and 48 HCs to study the differences in global and nodal topological properties among the three groups. In addition, the relationships between those significant network measures and cognitive function were further analyzed.

## 2 METHODS

This prospective study was approved by the institutional review board and followed the ethical guidelines of the Declaration of Helsinki, and written informed consent was acquired from each subject before inclusion.

### 2.1 Participants

From July 2018 to January 2020, a total of 66 patients with ESRD were included in our study and were divided into CI group (36 patients, 23 males and 13 females; mean age 31.19 ± 7.90 years, range from 19 to 42 years) and non‐CI (NCI) group (30 patients, 20 males and 10 females; mean age 31.33 ± 7.09 years, range from 19 to 45 years). Patients were included if they 1) had chronic glomerulonephritis with a disease duration of greater than 6 months for all patients with ESRD; 2) had an age of greater than or equal to 18 years; and 3) had normal vision, were right‐handed, and were able to complete cognitive function assessments. The exclusion criteria were as follows: (a) history of drug abuse, or alcohol addiction; (b) any acute cardiovascular and cerebrovascular diseases, such as acute ischemic cerebrovascular disease, acute arterial embolism, and acute heart failure; (c) previous history of neurological disease, such as tumor, trauma, epilepsy, or Alzheimer's disease; (d) any history of psychiatric disorders; (e) history of diabetic nephropathy or hypertensive nephropathy; and (f) head movement greater than 1.0 mm or 1.0°.

In addition, 48 HCs (30 males and 18 females; mean age 31.93 ± 7.99 years, range from 18 to 42 years) were also recruited, with age, sex, and education level matched to those of the ESRD patients. There were no renal disease or other systemic diseases in the HC group. Other exclusion criteria were the same as those applied to ESRD patients.

### 2.2 Neuropsychological tests

The diagnosis of CI was based on the score of the Montreal Cognitive Assessment (MoCA). The patients were diagnosed with CI with a MoCA total score < 26 (Nasreddine et al., 2005). All subjects also need to complete the Trail Making Test A (TMT‐A), Trail Making Test B (TMT‐B), and Symbol Digit Modalities Test (SDMT). All neuropsychological tests were assessed by a neurologist with 20 years of experience before MR scanning.

### 2.3 Laboratory examinations

Blood and urine tests were performed for all patients with ESRD within 24 hr before MRI examinations, including those to measure hemoglobin, hematocrit, serum calcium, total cholesterol, triglycerides, high‐density lipoprotein cholesterol, low‐density lipoprotein cholesterol, serum creatinine, blood uric acid, and blood urea nitrogen levels. No biochemistry tests were performed for HCs.

### 2.4 Image acquisition

All the MR scans were performed on a 3.0‐T MR scanner (Discovery 750 system; GE Healthcare, Waukesha, WI) with a 16‐channel head coil. Subjects were placed in a supine position with a foam pad fixed on the head to reduce head motion during MR scanning. At the same time, rubber earplugs were used to minimize the discomfort that the noise caused to the subjects. During MR scanning, all subjects were told to be quiet, keep their eyes closed and heads still, but stay awake, and try not to think about anything. The Rs‐fMRI data were obtained using a gradient‐echo echo‐planar (GRE‐EPI) sequence with the following parameters: 32 axial slices; repetition time/echo time, 2000/41 ms; field of view, 220 × 220 mm^2^; matrix, 64 × 64; section thickness, 5 mm; slice gap, 0.4 mm; flip angle, 90°. Each functional scan included 240 brain volumes and lasted 480 s. High‐resolution three‐dimensional T_1_‐weighted anatomical images were acquired using a three‐dimensional brain volume imaging (3D‐BRAVO) sequence (188 sagittal slices, 1‐mm in‐plane resolution).

### 2.5 Data preprocessing

Data preprocessing was performed for all Rs‐fMRI data using software (Data Processing & Analysis for Brain Imaging toolbox; http://rfmri.org/DPABI) (Yan et al., 2016). The detailed steps were as follows: (a) the first 10 volumes were removed to ameliorate the possible effects of imager instability and subjects’ adaptation to the MRI environment; (b) the slice timing and realignment were performed; (c) the images were spatially normalized to the standard Montreal Neurological Institute (MNI152) space (resampling voxel size = 3 × 3 × 3 mm^3^) through Diffeomorphic Anatomical Registration Through Exponentiated Lie Algebra (DARTEL) (Ashburner, 2007); (d) the spatial smoothing was performed with a 4‐mm full width at half‐maximum Gaussian kernel; (e) the BOLD signals were detrended for the removal of linear trends; (f) temporal filtering (bandpass, 0.01–0.08 Hz); (g) regression of nuisance signals (cerebrospinal fluid signals, white matter signals, and Friston‐24 head motion parameters); and (h) the scrubbing was performed to delete motion volumes.

### 2.6 Network construction

#### 2.6.1 Node definition

A large‐scale functional network was constructed for each participant using freely available software (GRaph thEoreTical Network Analysis toolbox; www.nitrc.org/projects/GRETNA) (J. Wang et al., 2015). A network was constructed with edges representing connections among nodes and with nodes representing brain regions. The nodes of the functional brain networks were defined according to the automated anatomical labeling atlas (Tzourio‐Mazoyer et al., 2002), which is the most widely used atlas in graph theoretical analysis. Using this atlas, the whole brain was divided into 90 anatomical regions including 78 cortical regions and 12 subcortical regions. Each region was regarded as a network node.

#### 2.6.2 Edge definition

To define the network edges, we extracted the average temporal series of all voxels within each region. Then, we calculated the partial correlation coefficients between the regional average temporal series for each pair of nodes, correcting for the effects of the remaining 88 regions (representing their conditional dependences). Thus, a 90 × 90 partial correlation matrix was created for each subject. Finally, individual partial correlation matrices were converted into binarized matrices based on a predefined threshold (described below for the threshold selection) (J. Zhang et al., 2011).

### 2.7 Network analysis

#### 2.7.1 Threshold selection

We applied a wide range of sparsity thresholds (*S*) to all correlation matrices. The minimum and maximum values of *S* used were established, ensuring that the thresholded networks were estimable for the small‐worldness scalar *σ*, and that the *σ* was larger than 1.0 for all participants (J. Zhang et al., 2011).

#### 2.7.2 Network metrics

Definition of these network properties have been described in previous studies (Q. Wang et al., 2012; J. Zhang et al., 2011), and a brief interpretation of these network properties has been summarized in our previous study (Wu et al., 2020). For brain functional networks at each sparsity threshold, we calculated the topological properties of brain functional networks at both global and nodal levels. The global topological properties included: 1) small‐world parameters comprising characteristic path length (*L_p_*), clustering coefficient (*C_p_*), normalized characteristic path length (*λ*), normalized clustering coefficient (*γ*), and small‐worldness (*σ*); and 2) network efficiency comprising global efficiency (*E_glob_*) and local efficiency (*E_loc_*). The nodal‐level properties included three nodal centrality metrics: the degree, efficiency and betweenness. Brain regions showing significant between‐group differences in at least one nodal metric were identified.

### 2.8 Statistical analysis

#### 2.8.1 Differences in demographic and clinical variables

Statistical analysis was performed by using software (SPSS version 21.0, IBM Corp, Armonk, NY). The chi‐square test was used to compare gender ratio differences among the three groups. An analysis of variance (ANOVA) or independent two‐sample *t* test was used to determine the group differences of quantitative variables. If the ANOVA test found significant differences, a post hoc test was performed to assess the intergroup differences.

#### 2.8.2 Differences in network metrics

We calculated the area under the curve of all network metrics over the whole range of sparsity thresholds which provided a summarized scalar for topological characterization of brain networks independent of a single threshold selection. This approach enabled the exploration of between‐group differences in relative network organization, which is sensitive to detecting topological alterations of brain disorders (J. Zhang et al., 2011).

Group differences of network metrics were tested using an analysis of covariance (ANCOVA), and a post hoc test was performed if the ANCOVA showed significant differences. The potential confounding factors, including age, sex, and glomerular filtration rate, were set as nuisance covariates. Statistical significance was defined as *p* <.05.

#### 2.8.3 Correlation analysis

Exploratory partial correlation analysis was performed to examine the relationships between those significant network measures and neuropsychological test results and serum creatinine, serum urea, and hemoglobin levels in the CI group. The *p* values were corrected by the Benjamini–Hochberg false discovery rate (FDR). An FDR *q* value < 0.05 was considered statistically significant (Genovese et al., 2002).

## 3 RESULTS

### 3.1 Demographics and clinical characteristics

Table 1 summarizes the demographic and clinical data for patients with ESRD and HCs. There were no significant differences in age (*p* =.896), sex (*p* =.933) or education level (*p* =.476) among the three groups. There were significant differences in all neuropsychological test results among the three groups, and pair‐wise comparisons showed that the CI group performed worse compared with the NCI group and HC group (all *p* <.05). No significant differences were found in cognitive function between the NCI group and HC group (all *p* >.05). Regarding all the results of blood biochemistry tests, only hemoglobin levels in the CI group were found to be significantly lower than those in the NCI group (*p* <.05).

**TABLE 1:** Demographic and clinical data of the three groups

Parameters	CI (*n* = 36)	NCI (*n* = 30)	HC (*n* = 48)	*p* value
Demographic factors				
Sex (male/female)	23/13	20/10	30/18	.933 ^a^
Age (years)	31.19 ± 7.90	31.33 ± 7.09	31.93 ± 7.99	.896 ^c^
Education (years)	11.47 ± 2.26	10.80 ± 2.27	11.60 ± 3.62	.476 ^c^
Disease duration (months)	30.42 ± 9.63	29.77 ± 7.95	‐	.769 ^b^
Dialysis duration (months)	13.50 ± 3.33	12.17 ± 2.95	‐	.093 ^b^
Clinical characteristics				
BMI (kg/m^2^)	21.77 ± 3.60	22.16 ± 3.10	22.63 ± 2.56	.438 ^c^
SBP (mm Hg)	140.44 ± 17.88	141.77 ± 17.29	124.17 ± 10.19	<.001 ^c^
DBP (mm Hg)	86.11 ± 9.25	86.60 ± 12.27	78.60 ± 9.56	.001 ^c^
HDL‐C (mmol/L)	1.02 ± 0.30	1.01 ± 0.33	‐	.946 ^b^
LDL‐C (mmol/L)	2.25 ± 0.66	2.33 ± 0.90	‐	.698 ^b^
Total cholesterol (mmol/L)	3.96 ± 0.99	4.48 ± 1.64	‐	.136 ^b^
Triglycerides (mmol/L)	1.72 ± 0.91	2.23 ± 1.37	‐	.088 ^b^
Hemoglobin (g/L)	83.66 ± 17.52	95.22 ± 22.78	‐	.027 ^b^
Hematocrit (%)	27.81 ± 7.52	29.07 ± 7.15	‐	.490 ^b^
Serum calcium (mmol/L)	2.08 ± 0.34	2.10 ± 0.28	‐	.798 ^b^
Kidney function‐related characteristics				
Serum creatinine (μmol/L)	764.40 ± 229.66	714.62 ± 182.45	‐	.340 ^b^
Urea (mmol/L)	19.83 ± 7.74	20.58 ± 8.08	‐	.704 ^b^
Uric acid (μmol/L)	428.42 ± 117.29	405.82 ± 84.22	‐	.381 ^b^
Neuropsychological tests				
MoCA (score)	21.00 ± 4.57	27.27 ± 0.91	27.54 ± 1.11	<.001 ^c^
TMT‐A (sec)	51.69 ± 16.56	43.23 ± 13.27	38.19 ± 9.32	<.001 ^c^
TMT‐B (sec)	71.42 ± 16.50	63.17 ± 12.64	58.19 ± 9.83	<.001 ^c^
SDMT (score)	37.03 ± 11.24	44.50 ± 10.28	48.21 ± 10.15	<.001 ^c^

Abbreviations: BMI, body mass index; CI, cognitive impairment; DBP, diastolic pressure; HC, healthy control subjects; HDL‐C, high‐density lipoprotein cholesterol; LDL‐C, low‐density lipoprotein cholesterol; MoCA, Montreal Cognitive Assessment; NCI, noncognitive impairment; SBP, systolic pressure; SDMT, Symbol Digit Modalities Test; TMT‐A, Trail Making Test A; TMT‐B, Trail Making Test B.

All quantitative data are expressed as mean ± standard deviation; numbers for sex data.

^a^ The *p* value was calculated by using chi‐square test.

^b^ The *p* value was calculated by using independent two‐sample *t* test.

^c^ The *p* value was calculated by one‐way analysis of variance (ANOVA).

### 3.2 Alterations in small‐world properties

**FIGURE 1:**
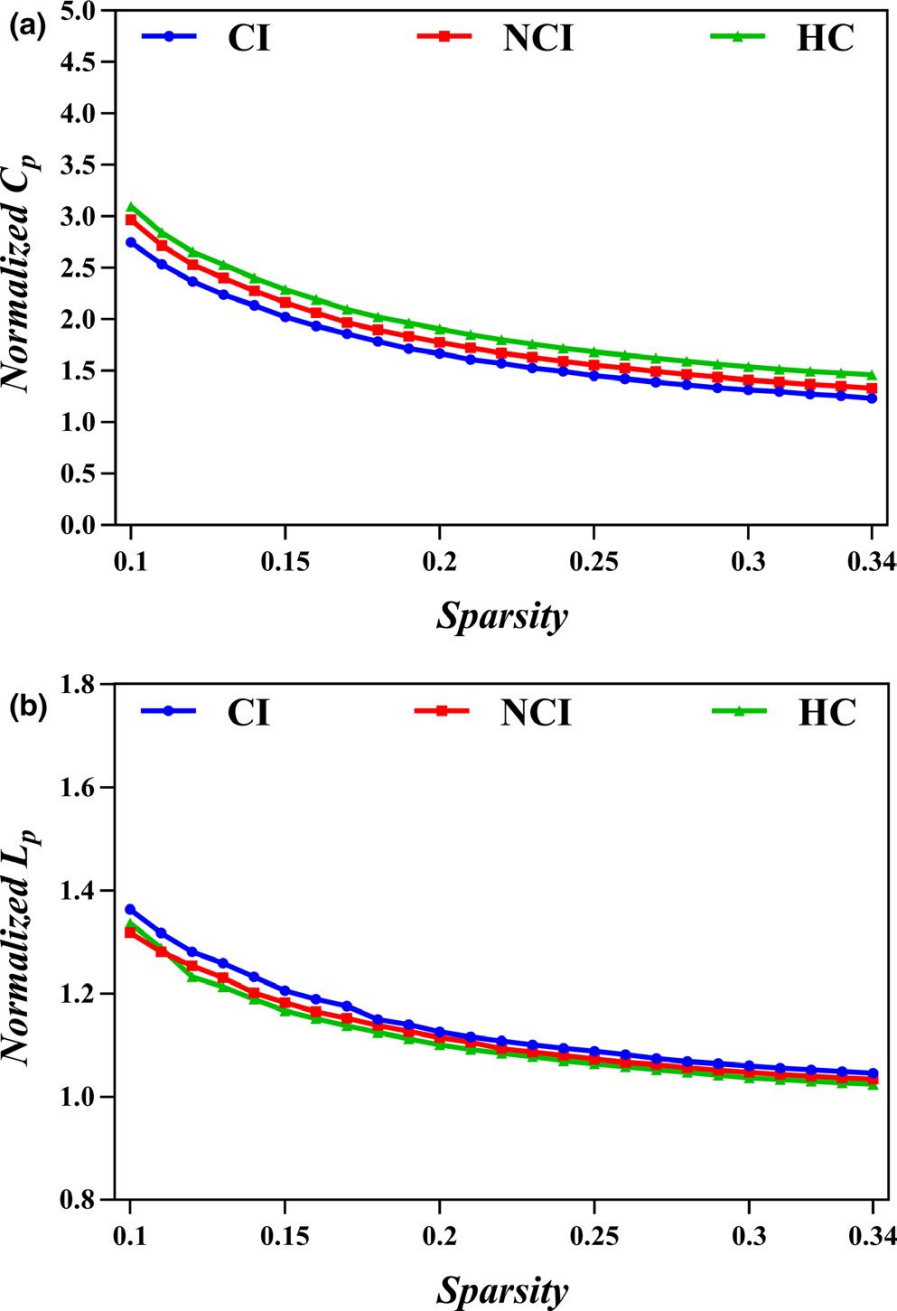
Small‐world properties in the functional brain networks are demonstrated in the three groups within the whole range of sparsity thresholds (0.1 < *S* < 0.34), with normalized clustering coefficient (*C_p_*) (*γ*) > 1 (a) and normalized characteristic path length (*L_p_*) (*λ*) ≈ 1 (b). Abbreviations: CI, cognitive impairment; HC, healthy control subjects, NCI, noncognitive impairment

**FIGURE 2:**
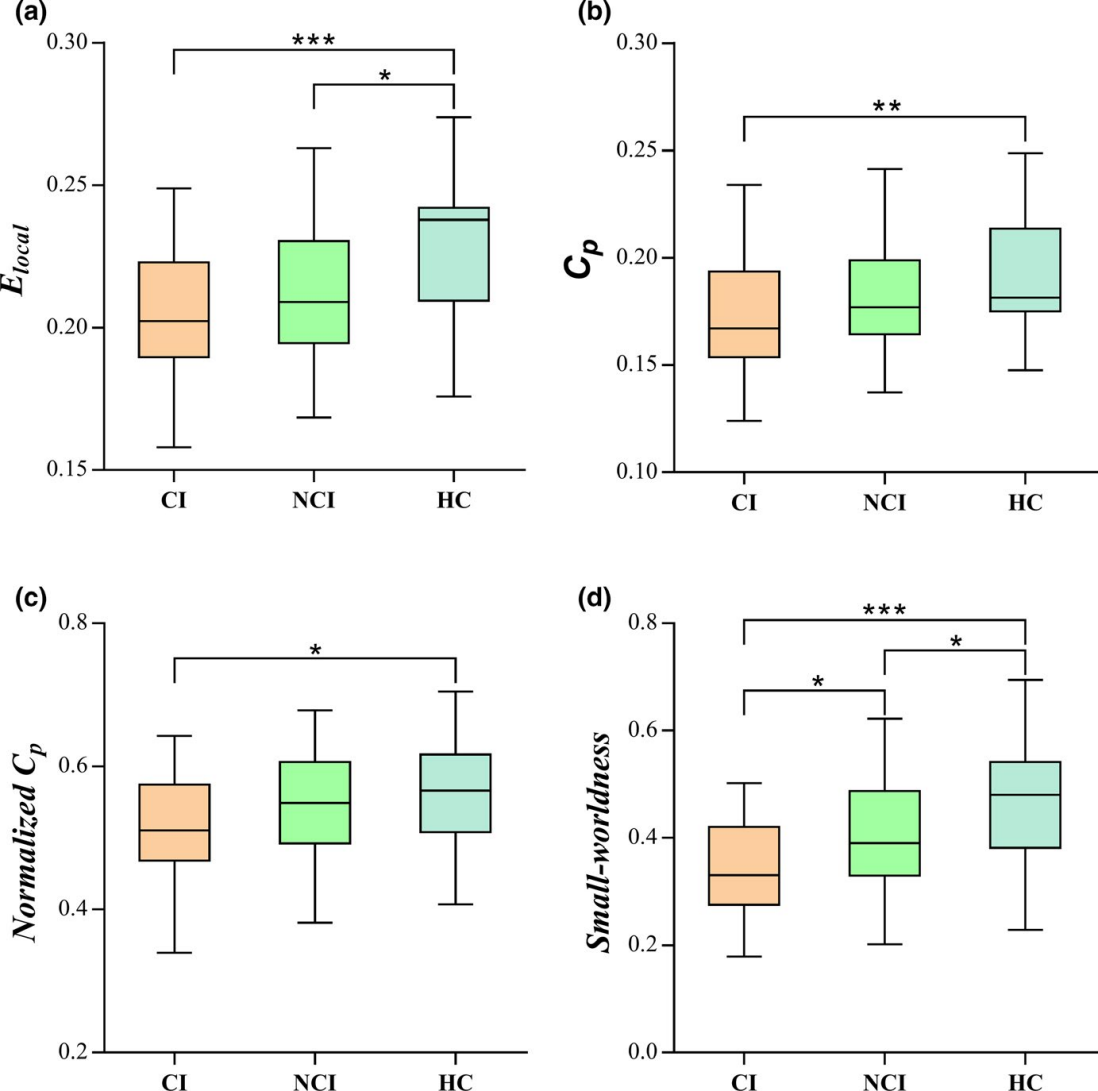
Comparisons of the three groups in global efficiency (*E_glob_*) (a), clustering coefficient (*C_p_*) (b), normalized clustering coefficient (*γ*) (c), and small‐worldness (*σ*) (d). ^*^ *p* < .05, ^**^ *p* < .01; ^***^ *p* < .001 (Bonferroni correction). Abbreviations: CI, cognitive impairment; HC, healthy control subjects; NCI, noncognitive impairment

Small‐world properties were demonstrated in ESRD patients with and without CI and HCs across the whole range of sparsity thresholds (0.1–0.34), with *γ* > 1, *λ* ≈ 1 (Figure 1). However, the small‐worldness *σ* progressively decreased from that in HCs to that of NCIs to that of CIs. The CI group exhibited lower clustering coefficient *C_p_* and normalized clustering coefficient *γ* than the HC group (*p* <.05). There were no significant differences in both the characteristic path length *L_p_* and normalized characteristic path length *λ* (*p* >.05) among the three groups. With regard to network efficiency, patients in both the CI group and NCI group exhibited lower local efficiency *E_loc_* (*p* <.05) but nonsignificant change of global efficiency *E_glob_* (*p* >.05) compared with HCs (Table 2 and Figure 2).

**TABLE 2:** Global topological parameters of brain functional networks in participants of the three groups

Network measures	CI (*n* = 36)	NCI (*n* = 30)	HC (*n* = 48)	ANCOVA	Post hoc analysis		
					CI versus HC	NCI versus HC	CI versus NCI
				*p* (*f*) value	*p* value	*p* value	*p* value
*E_glob_*	0.165 ± 0.010	0.167 ± 0.008	0.169 ± 0.006	0.062 (2.844)^a^	‐	‐	‐
*E_loc_*	0.205 ± 0.022	0.212 ± 0.024	0.228 ± 0.022	<0.001 (11.067)^a^	<.001^b^	.011^b^	.625
*C_p_*	0.173 ± 0.027	0.182 ± 0.025	0.192 ± 0.027	0.007 (5.259)^a^	.005^b^	.335	.521
*L_p_*	0.568 ± 0.052	0.555 ± 0.035	0.549 ± 0.033	0.102 (2.327)	‐	‐	‐
*γ*	0.518 ± 0.076	0.542 ± 0.074	0.564 ± 0.072	0.023 (3.916)^a^	.018^b^	.608	.605
*λ*	0.332 ± 0.019	0.328 ± 0.015	0.327 ± 0.013	0.346 (1.071)	‐	‐	‐
*σ*	0.342 ± 0.082	0.405 ± 0.098	0.469 ± 0.111	<0.001 (16.981)^a^	<.001^b^	.020^b^	.034^b^

Abbreviations: CI, cognitive impairment; HC, healthy control subjects; NCI, noncognitive impairment.

Global network measures of brain functional network are area under the curve of the network properties across the full range of sparsity thresholds (0.1–0.34).

^a^ *p* < 0.05 calculated by using analysis of covariance (ANCOVA).

^b^ *p* < 0.05 after post hoc analysis (Bonferroni correction).

### 3.3 Alterations in regional nodal characteristics

Compared to HCs, patients in both the CI group and the NCI group displayed significant decreased nodal centralities in the bilateral medial part of the superior frontal gyrus (SFGmed), left posterior cingulate gyrus (PCG), left angular gyrus (ANG), right precuneus (PCUN), right amygdala (AMYG), and right hippocampus (HIP). Compared with patients in the NCI group, those in the CI group exhibited significant decreased nodal centralities in the bilateral SFGmed, right PCUN, and left PCG (Table 3).

**TABLE 3:** Regions showing abnormal nodal centralities in ESRD patients compared with healthy control subjects

Brain regions	Functional classification	*p* value		
		Nodal degree	Nodal efficiency	Nodal betweenness
CI < healthy control subjects				
Right superior frontal gyrus, medial	Association	<.05	<.05	<.05
Left superior frontal gyrus, medial	Association	<.05	<.05	.137
Left posterior cingulate gyrus	Paralimbic	<.05	<.05	<.05
Right anterior cingulate and paracingulate gyri	Paralimbic	<.05	<.05	.107
Left angular gyrus	Association	<.05	<.05	.223
Right precuneus	Association	<.05	.067	.281
Right precentral gyrus	Primary	<.05	<.05	.115
Right amygdala	Subcortical	<.05	<.05	.321
Right hippocampus	Limbic	<.05	<.05	<.05
NCI < healthy control subjects				
Right superior frontal gyrus, medial	Association	<.05	<.05	<.05
Left superior frontal gyrus, medial	Association	<.05	<.05	<.05
Left posterior cingulate gyrus	Paralimbic	<.05	<.05	.172
Left angular gyrus	Association	<.05	0.078	.231
Right precuneus	Association	<.05	<.05	.082
Right amygdala	Subcortical	<.05	.104	.321
Right hippocampus	Limbic	<.05	<.05	<.05
CI < NCI				
Right superior frontal gyrus, medial	Association	<.05	<.05	.068
Left superior frontal gyrus, medial	Association	<.05	<.05	<.05
Right precuneus	Association	.054	<.05	.152
Left posterior cingulate gyrus	Paralimbic	<.05	<.05	<.05

Abbreviations: CI, cognitive impairment; NCI, noncognitiveimpairment.

### 3.4 Relationships between network measures and clinical variables

**FIGURE 3:**
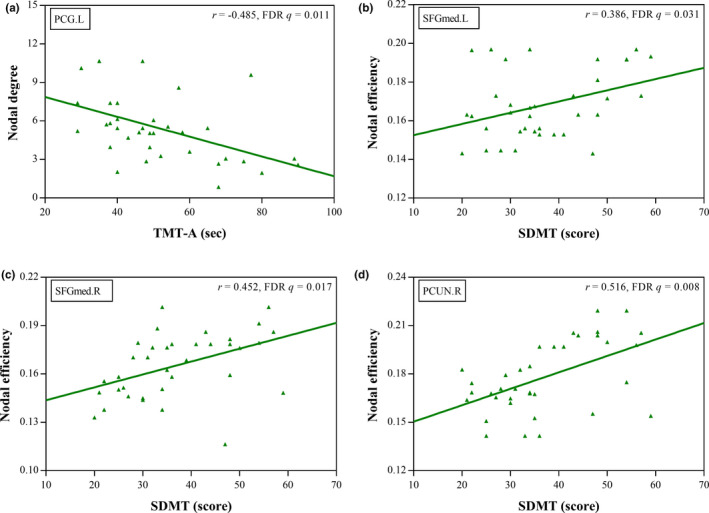
Scatter plots show partial correlations analyses of the network measures and neurocognitive test scores in ESRD patients with cognitive impairment. The completion time of Trail Making Test A (TMT‐A) was negatively related to nodal degree in the left posterior cingulate gyrus (PCG.L) (a). The Symbol Digit Modalities Test (SDMT) scores were positively associated with nodal efficiency in the bilateral medial part of the superior frontal gyrus (SFGmed) (b‐c) and right precuneus (PCUN.R) (d)

The partial correlation analysis results are shown in Figure 3. In the CI group, the completion time of TMT‐A was negatively correlated with nodal degree in the left PCG (*r* = −0.485, FDR *q* = 0.011). Moreover, SDMT scores were positively associated with nodal efficiency in the bilateral SFGmed (*r* = 0.386, FDR *q* = 0.031 and *r* = 0.452, FDR *q* = 0.017 for left and right, respectively) and right PCUN (*r* = 0.516, FDR *q* = 0.008). None of the significant network measures showed a correlation with MoCA scores, serum creatinine levels, serum urea levels, or hemoglobin levels of ESRD patients with CI (FDR *q* > 0.05).

## 4 DISCUSSION

By using Rs‐fMRI in combination with graph theoretical analysis, the results of the present study demonstrated alterations in brain functional networks in patients with ESRD. Four main findings were identified in our study. First, at a global level, the normal integration and segregation of brain functional networks in patients with ESRD were disrupted, as indicated by decreased local efficiency *E_loc_* and small‐worldness *σ* compared with HCs. Second, at the regional level, patients with ESRD showed significantly decreased nodal centralities in the DMN regions and right AMYG. Third, significant decreases in global (small‐worldness *σ*) and nodal (nodal centralities in the bilateral SFGmed, right PCUN, and left PCG) topological properties were found in the CI group compared to the NCI group, suggesting a much more severe disruption of brain functional networks in ESRD patients with CI. Fourth, altered nodal centralities in the left PCG, bilateral SFGmed, and right PCUN were associated with cognitive function in ESRD patients with CI, which establishes a clinical relevance of the observed neural network alterations. Consequently, these findings extend our understanding of the potential pathophysiology of CI in patients with ESRD from a network perspective.

Small‐world network organization provides a highly integrated and optimized network model for the best balance between the segregation and integration of information (Rubinov & Sporns, 2010). Small‐world architectures can not only support specific modular information and fast global information processing, but also maximize the efficiency of brain networks (He & Evans, 2010). Despite the common small‐world topology for both patients with ESRD and HCs, our findings of lower local efficiency *E_loc_* and small‐worldness *σ* in patients with ESRD compared with HCs revealed a decreased ability to transmit information at the local level and a “weaker small‐worldization” of brain functional networks in patients with ESRD (Latora & Marchiori, 2001). These findings indicate that the optimized balance of small‐world network organization has shifted in a high‐cost and low‐profit manner in patients with ESRD (Bullmore & Sporns, 2012). Moreover, a significant reduction in small‐worldness *σ* was observed in the CI group compared to the NCI group, suggesting that disrupted small‐world brain functional network was more severe in ESRD patients with CI. Consistent with our findings, a recent study demonstrated that ESRD patients who underwent HD exhibit disruptions in brain functional networks, as characterized by lower normalized clustering coefficient *γ*, small‐worldness *σ*, global efficiency *E_glob_*, and local efficiency *E_loc_* compared with control subjects (Jin et al., 2020).

In our study, both patient groups showed decreased nodal centralities mainly in the DMN regions, including the bilateral SFGmed, left PCG, left ANG, right PCUN, and right HIP (Buckner et al., 2008). ESRD‐related gray matter volume deficits (L. J. Zhang et al., 2013) and decreases in spontaneous brain activity (Luo et al., 2016) have been reported in several DMN regions, including the PCG, PCUN, and medial prefrontal cortex (MPFC) (i.e., bilateral SFGmed). Moreover, impaired functional connectivity in the DMN has also been demonstrated in patients with ESRD (Ni et al., 2014). Notably, a previous study using DTI in combination with graph theoretical analysis reported that ESRD patients with long‐term HD showed decreased node strength in the DMN regions, including the bilateral HIP, right parahippocampal gyrus, and right median cingulate and paracingulate gyri (DCG), as well as reduced nodal degree in the bilateral DCG (Chou et al., 2019). Moreover, a recent Rs‐fMRI study showed that patient with ESRD who received HD treatment exhibited decreased nodal centralities in some DMN regions, including the bilateral PCUN, right ANG, and right HIP (Jin et al., 2020). Thus, evidence from the previous studies provides further support for our findings.

Interestingly, the reduction in nodal centralities in the bilateral SFGmed, right PCUN, and left PCG we observed in our study was more pronounced in ESRD patients with CI than in those who had not CI, which suggests that nodal properties of the DMN regions may be sensitive imaging markers for detecting CI. Similarly, Ni et al. found a significant reduction in functional connectivity in the MPFC in patients with nonnephrotic encephalopathy (NE) compared to those with minimal NE (Ni et al., 2014). Specifically, the MPFC is a critical anterior hub of the DMN (Buckner et al., 2008) and has been posited to serve a variety of social, affective, and cognitive functions (Lieberman et al., 2019). Additionally, the PCG and PCUN are critical posterior hubs of the DMN (Buckner et al., 2008). The PCG plays an important role in cognitive function, which is involved in arousal and awareness, controlling the balance between internal and external attention, and environmental change detection. The PCUN is mainly responsible for highly integrated tasks including maintaining wakefulness and regulating visual–spatial episodic memory. Decreased nodal centralities in the MPFC, PCG, and PCU in ESRD patients with CI may result in several cognitive dysfunctions observed in the CI group, such as the deficits in attention, processing speed, executive function, motor function, and memory. Moreover, our further partial correlation analysis results of the CI group also support these findings partially, which revealed that the completion time of TMT‐A was negatively correlated with nodal degree in the left PCG, and SDMT scores were positively related to nodal efficiency in the bilateral SFGmed and right PCUN. Notably, TMT‐A and SDMT are neuropsychological tests that evaluate cognitive domains in concentration, mental tracking, visuomotor speed, psychomotor speed, attention, and visual memory (Cook et al., 2017).

We also found decreased nodal centralities in the right AMYG in patients with ESRD. The AMYG is mainly associated with emotion processing (Dalton et al., 2005). A previous study had demonstrated that clinical depression was prevalent in HD patients (Fan et al., 2014), and functional connectivity in the amygdala–prefrontal–posterior cingulate cortex–limbic circuits was damaged in depressive HD patients (Chen et al., 2017). Therefore, we speculate that decreased nodal centralities in the amygdala may be related to the depression complications in patients with ESRD patients. Unfortunately, we did not assess the emotional status of ESRD patients in this study, and this needs to be further investigated in our future studies.

We acknowledge several limitations in this study. First, it was a cross‐sectional study with a relatively small sample size, which may affect the power of the statistical analysis. Future studies with large sample sizes are needed. Second, we only applied the AAL 90 template to construct brain functional networks, but using different templates may affect the consistency of our results. Further studies are needed to determine which template is most appropriate for the characterization of network topology in patients with ESRD. Third, depression and anxiety are commonly reported among patients with ESRD undergoing HD (Semaan et al., 2018), but the patient's emotional state was not evaluated in the present study. In future studies, we should assess the depression and anxiety levels and their relationships with the changes of network metrics among patients with ESRD using appropriate neuropsychologic tests, such as Hamilton Depression Rating Scale and Hamilton Anxiety Rating Scale. Finally, we explored only the functional brain networks in patients with ESRD. There exist both anatomical and functional connections in the human brain. Therefore, future studies with multimodal techniques combining structural and functional MRI data will be helpful to provide more powerful evidence for the alterations in the topological properties of MDD patients and to interpret the relationship between the two modal networks.

## 5 CONCLUSIONS

In summary, our study investigated the topological architecture of brain functional networks in patients with ESRD who had CI using Rs‐fMRI data in combination with graph theory methods. Patients with ESRD showed lower local efficiency *E_loc_* and small‐worldness *σ* compared with HCs. In addition, regions with decreased nodal centralities were mainly distributed in the DMN in patients with ESRD. Moreover, comparison of the patient groups demonstrated significant reductions in some network metrics in the CI group compared to the NCI group. Importantly, decreased nodal centralities in the DMN regions, the bilateral SFGmed, left PCG, and right PCUN, might contribute to the reduced performance on neurocognitive tests in ESRD patients with CI. Our study suggests that the graph‐theory‐based network analysis provides a new perspective and potential imaging biomarkers for understanding the potential pathophysiological mechanisms of CI in patients with ESRD.

## CONFLICT OF INTEREST

All authors declare that there is no conflict of interest.

## AUTHOR CONTRIBUTIONS

BLW conceived and designed the study, supervised the conduct of the study, reviewed and revised the manuscript, and takes responsibility for the paper. ZY, PMW, and JPR were responsible for data acquisition. BLW, ZY, and PMW analyzed the data. XKL assisted with the literature review. ZY and PMW drafted the initial manuscript. All authors read and approved the final manuscript.

### PEER REVIEW

The peer review history for this article is available at https://publons.com/publon/10.1002/brb3.2076.

## Data Availability

The data that support the findings of this study are available from the corresponding author upon reasonable request.
